# Supplementary material for: A Likelihood Approach for Real-Time Calibration of Stochastic Compartmental Epidemic Models
Source: PLoS Comput Biol. 2017 Jan 17;13(1):e1005257. doi: 10.1371/journal.pcbi.1005257 (PMC5240920; doi:10.1371/journal.pcbi.1005257)
Supplement: S1 File — (TAR.GZ) [file pcbi.1005257.s014.tar.gz › HSPH_Online-SI-Revision/EnKF07/n100-extreme/n100-extreme_EnKF2_table-4.pdf]

1	newly 3 weeks spec	10683.	772.33	{13., 1381.}	92.7705	1.82528	newly 3 weeks cumul	25079.	5209.39	{126., 14009.}	87.2172	1.27759	newly 5 weeks spec	10855.	309.17	{0., 600.}	97.1518	2.62281	newly 5 weeks cumul	47373.	6000.	{161., 16681.}	88.299	1.46011	newly 58 weeks cumul 85292. 6842.89 {210., 18280.} 91.9771 1.65401
2	newly 3 weeks spec	8383.	901.93	{1., 893.}	94.6677	1.87444	newly 3 weeks cumul	21608.	11880.8	{2093., 12056.}	103.655	0.776981	newly 5 weeks spec	8027.	231.01	{0., 230.}	98.2717	2.74318	newly 5 weeks cumul	38163.	12571.1	{2152., 12264.}	95.637	0.961762	newly 54 weeks cumul 72415. 12790.4 {2166., 12283.} 90.1929 1.18719
3	newly 3 weeks spec	12915.	1126.07	{7., 1150.}	91.6533	1.93434	newly 3 weeks cumul	29820.	5811.28	{53., 4010.}	93.3183	1.45913	newly 5 weeks spec	11421.	328.18	{1., 694.}	97.1265	2.57238	newly 5 weeks cumul	54424.	6731.88	{61., 5620.}	91.2142	1.64462	newly 59 weeks cumul 84212. 7449.17 {65., 9877.} 91.1543 1.7927
4	newly 3 weeks spec	15590.	2310.1	{2., 8107.}	86.6885	2.03846	newly 3 weeks cumul	27674.	34271.4	{4872., 74464.}	75.0146	0.347672	newly 5 weeks spec	15345.	893.42	{0., 2979.}	94.1778	3.15867	newly 5 weeks cumul	61225.	36494.6	{6137., 74464.}	59.726	0.477842	newly 63 weeks cumul 88766. 39043.9 {7244., 74994.} 62.02 0.566514
5	newly 3 weeks spec	10521.	2721.28	{0., 5093.}	100.904	2.21546	newly 3 weeks cumul	21016.	8535.67	{939., 44498.}	90.3635	0.948413	newly 5 weeks spec	12782.	1086.65	{0., 8911.}	91.4986	3.00231	newly 5 weeks cumul	46785.	11678.2	{941., 51478.}	87.7199	1.20156	newly 60 weeks cumul 85329. 12890.9 {941., 62850.} 84.8927 1.39898
6	newly 3 weeks spec	10702.	860.66	{10., 1369.}	91.958	1.72924	newly 3 weeks cumul	23963.	5683.24	{120., 13453.}	85.5526	1.14654	newly 5 weeks spec	11271.	368.01	{0., 585.}	96.7349	2.42533	newly 5 weeks cumul	47247.	6597.62	{147., 16064.}	87.0746	1.34599	newly 58 weeks cumul 86171. 7645.89 {153., 17643.} 91.1271 1.54702
7	newly 3 weeks spec	11555.	1433.59	{45., 1578.}	92.4441	1.6766	newly 3 weeks cumul	26529.	9583.52	{278., 10439.}	95.5612	1.03264	newly 5 weeks spec	11237.	544.05	{2., 1016.}	95.1584	2.5434	newly 5 weeks cumul	49918.	10987.	{331., 10992.}	92.7129	1.23462	newly 56 weeks cumul 84780. 12507.2 {355., 11483.} 90.1577 1.40494
8	newly 3 weeks spec	11018.	862.91	{14., 1402.}	92.1682	1.74317	newly 3 weeks cumul	24447.	5670.38	{140., 13565.}	85.6348	1.15438	newly 5 weeks spec	11207.	366.25	{0., 578.}	96.732	2.39371	newly 5 weeks cumul	47781.	6579.07	{146., 16076.}	87.2372	1.35239	newly 58 weeks cumul 87124. 7610.32 {157., 17601.} 91.265 1.55648
9	newly 3 weeks spec	10870.	879.13	{36., 1371.}	91.9123	1.52553	newly 3 weeks cumul	22125.	6134.73	{258., 11541.}	83.7854	0.929767	newly 5 weeks spec	12694.	375.08	{3., 618.}	97.0452	2.32458	newly 5 weeks cumul	47679.	7067.85	{320., 15377.}	86.2782	1.16387	newly 58 weeks cumul 88989. 8162.07 {386., 16809.} 90.828 1.3767
10	newly 3 weeks spec	15442.	1361.77	{250., 3313.}	91.1814	1.3015	newly 3 weeks cumul	29673.	24835.6	{7277., 67772.}	73.7062	0.416093	newly 5 weeks spec	11765.	264.41	{7., 301.}	97.7526	2.38695	newly 5 weeks cumul	57244.	25631.1	{8759., 68044.}	67.6202	0.574293	newly 62 weeks cumul 72720. 26032.1 {8828., 68112.} 69.6704 0.649
11	newly 3 weeks spec	9197.	2253.05	{45., 4605.}	93.3547	1.44561	newly 3 weeks cumul	23145.	12364.9	{721., 13491.}	101.264	0.905711	newly 5 weeks spec	8893.	649.42	{2., 1306.}	92.6974	2.24249	newly 5 weeks cumul	41559.	14184.4	{752., 22900.}	93.4978	1.07364	newly 56 weeks cumul 75767. 15584.3 {752., 40811.} 87.645 1.25978
12	newly 3 weeks spec	11654.	1269.85	{114., 1142.}	92.143	1.43324	newly 3 weeks cumul	24296.	6004.13	{723., 4572.}	96.621	1.10244	newly 5 weeks spec	12745.	551.93	{28., 705.}	95.6694	1.85791	newly 5 weeks cumul	50388.	7340.69	{856., 6129.}	93.0794	1.28722	newly 57 weeks cumul 88506. 10641.5 {958., 29314.} 89.4928 1.37629
13	newly 3 weeks spec	8371.	758.1	{0., 1000.}	95.5064	2.91191	newly 3 weeks cumul	19985.	10338.7	{1797., 11832.}	79.0135	0.618085	newly 5 weeks spec	8948.	197.78	{0., 565.}	97.7897	3.24436	newly 5 weeks cumul	38014.	10890.5	{1907., 11859.}	82.3498	0.819419	newly 55 weeks cumul 83218. 11664.8 {1928., 11868.} 86.7704 1.07976
14	newly 3 weeks spec	11434.	3517.61	{0., 25720.}	98.9639	2.22003	newly 3 weeks cumul	18644.	10150.	{936., 45427.}	96.6809	0.932205	newly 5 weeks spec	15310.	1397.64	{0., 9129.}	90.8711	2.88801	newly 5 weeks cumul	50732.	14177.6	{936., 75220.}	86.1707	1.22773	newly 60 weeks cumul 70240. 15777. {936., 81288.} 82.3044 1.31634
15	newly 3 weeks spec	13412.	2727.54	{0., 5085.}	96.9574	2.37243	newly 3 weeks cumul	27516.	8685.72	{940., 44842.}	86.1185	1.03045	newly 5 weeks spec	12056.	1080.8	{0., 8998.}	91.0352	3.00213	newly 5 weeks cumul	54113.	11810.1	{940., 51090.}	86.2611	1.24845	newly 60 weeks cumul 75951. 13000.6 {940., 62680.} 84.3239 1.35322
16	newly 3 weeks spec	15726.	2712.74	{0., 5095.}	94.6106	2.39398	newly 3 weeks cumul	30526.	8535.99	{941., 44452.}	85.6154	1.06648	newly 5 weeks spec	14011.	1099.43	{0., 9105.}	92.1531	3.01205	newly 5 weeks cumul	61514.	11699.5	{944., 51158.}	85.6764	1.29313	newly 60 weeks cumul 88752. 12915.2 {944., 63375.} 85.448 1.41403
17	newly 3 weeks spec	9053.	773.53	{0., 1028.}	95.3608	2.88653	newly 3 weeks cumul	21971.	9977.88	{1672., 11717.}	81.3203	0.708255	newly 5 weeks spec	9419.	209.94	{0., 607.}	97.7711	3.24265	newly 5 weeks cumul	40887.	10548.6	{1786., 11745.}	83.8174	0.900651	newly 55 weeks cumul 79924. 11402.6 {1805., 11836.} 86.8037 1.10879
18	newly 3 weeks spec	8959.	1428.46	{39., 1536.}	91.3948	1.45959	newly 3 weeks cumul	19558.	9941.18	{542., 10197.}	90.3156	0.781638	newly 5 weeks spec	10392.	502.64	{0., 954.}	95.1632	2.51695	newly 5 weeks cumul	40376.	11268.3	{577., 10835.}	90.0729	1.02378	newly 56 weeks cumul 75084. 12812.6 {594., 11113.} 89.0655 1.22622
19	newly 3 weeks spec	11412.	876.99	{52., 1513.}	92.3152	1.57277	newly 3 weeks cumul	23492.	6122.28	{301., 12619.}	84.0291	0.977342	newly 5 weeks spec	12862.	368.38	{3., 616.}	97.1359	2.35066	newly 5 weeks cumul	49674.	7039.73	{402., 15573.}	86.8087	1.20463	newly 58 weeks cumul 89565. 8109.67 {486., 17052.} 90.9455 1.4004
20	newly 3 weeks spec	15824.	2683.42	{3., 8293.}	83.5331	1.7702	newly 3 weeks cumul	21990.	37532.2	{7102., 101585.}	110.181	0.384478	newly 5 weeks spec	20039.	805.15	{0., 2924.}	95.9821	3.08955	newly 5 weeks cumul	66304.	39693.1	{7342., 105351.}	61.1658	0.502313	newly 63 weeks cumul 91232. 41438. {7388., 114581.} 62.5161 0.578852
21	newly 3 weeks spec	11565.	2709.39	{0., 4892.}	99.3312	2.33166	newly 3 weeks cumul	23774.	8642.36	{928., 44681.}	88.0273	0.988913	newly 5 weeks spec	12361.	1086.91	{0., 9136.}	91.2069	3.09844	newly 5 weeks cumul	49979.	11782.5	{932., 51189.}	86.9225	1.22766	newly 60 weeks cumul 80337. 12971.8 {932., 62674.} 84.1435 1.37964
22	newly 3 weeks spec	13043.	231.86	{0., 553.}	98.2223	2.48863	newly 3 weeks cumul	25673.	6925.33	{1013., 17853.}	78.3657	0.809778	newly 5 weeks spec	13126.	45.58	{0., 93.}	99.6528	3.46812	newly 5 weeks cumul	53143.	7067.19	{1127., 17858.}	87.4984	1.09819	newly 61 weeks cumul 81637. 7129.9 {1168., 17858.} 91.2663 1.27648
23	newly 3 weeks spec	8971.	819.21	{3., 620.}	95.2461	1.997	newly 3 weeks cumul	22691.	15022.6	{2013., 40064.}	116.047	0.844075	newly 5 weeks spec	8717.	207.13	{0., 202.}	98.4668	2.97936	newly 5 weeks cumul	40575.	15639.3	{2091., 72608.}	99.8992	1.02142	newly 54 weeks cumul 74886. 15836.8 {2100., 81014.} 89.9643 1.22238
24	newly 3 weeks spec	9753.	1142.66	{1., 1133.}	95.766	2.08672	newly 3 weeks cumul	22644.	19866.2	{1267., 104238.}	129.128	0.861599	newly 5 weeks spec	10377.	218.2	{0., 214.}	98.1509	3.17203	newly 5 weeks cumul	43672.	20578.2	{1267., 104238.}	101.406	1.04404	newly 54 weeks cumul 86096. 20772.7 {1267., 104238.} 86.1117 1.2451
25	newly 3 weeks spec	14475.	1235.05	{90., 1147.}	92.3972	1.55687	newly 3 weeks cumul	28620.	5880.98	{439., 4543.}	96.4276	1.20038	newly 5 weeks spec	13481.	546.86	{23., 625.}	95.9435	1.91368	newly 5 weeks cumul	57956.	7199.93	{555., 6055.}	93.2443	1.37818	newly 57 weeks cumul 86463. 10262.9 {646., 7593.} 89.8237 1.414
26	newly 3 weeks spec	8715.	767.25	{0., 989.}	95.4071	2.88305	newly 3 weeks cumul	21621.	9395.05	{273., 11572.}	83.3069	0.807277	newly 5 weeks spec	8698.	202.24	{0., 617.}	97.6749	3.2109	newly 5 weeks cumul	39423.	9950.23	{273., 11572.}	84.7207	0.99284	newly 55 weeks cumul 81661. 10779.7 {273., 11612.} 87.7102 1.22662
27	newly 3 weeks spec	9364.	2315.67	{60., 5732.}	92.5087	1.42739	newly 3 weeks cumul	24919.	12936.	{605., 17637.}	100.961	0.94266	newly 5 weeks spec	8776.	651.95	{4., 1256.}	92.5712	2.11226	newly 5 weeks cumul	43318.	14761.8	{743., 32804.}	93.2321	1.0966	newly 56 weeks cumul 79621. 16077.6 {835., 71472.} 86.8778 1.28307
28	newly 3 weeks spec	11755.	2702.58	{0., 5064.}	99.202	2.50142	newly 3 weeks cumul	27332.	8694.68	{844., 45064.}	86.4363	1.05547	newly 5 weeks spec	11093.	1052.05	{0., 8839.}	90.5161	3.13575	newly 5 weeks cumul	50746.	11756.	{844., 50537.}	86.8189	1.25697	newly 60 weeks cumul 87654. 12881.7 {844., 61716.} 85.3039 1.44365
29	newly 3 weeks spec	8104.	849.9	{4., 667.}	95.2273	1.87285	newly 3 weeks cumul	20099.	11458.6	{1724., 15352.}	106.67	0.799609	newly 5 weeks spec	8778.	203.94	{0., 208.}	98.4347	2.955	newly 5 weeks cumul	37699.	12080.3	{1724., 15879.}	96.7278	1.01005	newly 54 weeks cumul 75236. 12274.3 {1724., 15919.} 90.4647 1.25602
30	newly 3 weeks spec	13462.	2254.88	{0., 7858.}	85.3008	2.14834	newly 3 weeks cumul	25138.	35095.9	{4926., 73849.}	85.8351	0.354826	newly 5 weeks spec	14527.	855.1	{0., 2891.}	94.1137	3.25116	newly 5 weeks cumul	55394.	37240.6	{6057., 73849.}	58.4366	0.443507	newly 63 weeks cumul 91285. 39505. {7182., 74550.} 62.1055 0.572821
31	newly 3 weeks spec	13467.	7018.63	{959., 20710.}	66.7397	0.603973	newly 3 weeks cumul	21982.	45524.7	{12239., 96304.}	132.645	0.331733	newly 5 weeks spec	17282.	1799.83	{84., 3657.}	89.5855	1.38562	newly 5 weeks cumul	58272.	50784.7	{13881., 106689.}	63.2748	0.361744	newly 64 weeks cumul 87865. 53592.1 {14126., 107096.} 54.2708 0.404602
32	newly 3 weeks spec	11691.	228.74	{0., 548.}	98.0435	2.44931	newly 3 weeks cumul	23586.	6903.2	{1021., 17812.}	76.9142	0.776155	newly 5 weeks spec	12032.	45.16	{0., 100.}	99.6247	3.44098	newly 5 weeks cumul	48919.	7045.06	{1149., 17819.}	86.65	1.06477	newly 61 weeks cumul 74173. 7107.89 {1173., 17819.} 90.4297 1.23666
33	newly 3 weeks spec	9366.	1322.62	{104., 1912.}	91.6863	1.529	newly 3 weeks cumul	20595.	6691.67	{1083., 7158.}	95.9876	1.04916	newly 5 weeks spec	10722.	365.76	{8., 1011.}	96.5887	2.1808	newly 5 weeks cumul	42102.	7731.73	{1423., 9917.}	90.811	1.26491	newly 59 weeks cumul 87634. 8497.97 {1464., 13437.} 90.3029 1.52351
34	newly 3 weeks spec	6522.	969.81	{3., 894.}	95.0486	1.72069	newly 3 weeks cumul	16514.	12255.8	{1795., 18890.}	117.726	0.73736	newly 5 weeks spec	7135.	226.76	{0., 241.}	98.2669	2.7387	newly 5 weeks cumul	30872.	12951.6	{1812., 19767.}	101.539	0.934691	newly 54 weeks cumul 67088. 13162.1 {1812., 19844.} 91.0703 1.20358
35	newly 3 weeks spec	10120.	1344.29	{130., 2078.}	91.3112	1.45642	newly 3 weeks cumul	20852.	6875.52	{1312., 7316.}	95.3625	0.935306	newly 5 weeks spec	12158.	370.53	{11., 809.}	96.9524	2.21882	newly 5 weeks cumul	45344.	7929.9	{1460., 10169.}	90.1671	1.17841	newly 59 weeks cumul 90553. 8688.16 {1522., 13742.} 90.4054 1.42318
36	newly 3 weeks spec	8518.	1301.45	{292., 1068.}	90.2807	1.15011	newly 3 weeks cumul	20967.	6302.5	{1626., 4483.}	94.1551	0.885451	newly 5 weeks spec	8858.	584.29	{83., 658.}	93.4038	1.53007	newly 5 weeks cumul	39044.	7703.36	{2082., 5632.}	91.2437	1.03006	newly 57 weeks cumul 75101. 10721.8 {2597., 7787.} 88.906 1.17351
37	newly 3 weeks spec	14436.	1344.25	{246., 3268.}	90.6882	1.27716	newly 3 weeks cumul	27190.	24688.9	{6863., 67814.}	76.84	0.406402	newly 5 weeks spec	14344.	293.86	{5., 306.}	97.9513	2.5202	newly 5 weeks cumul	57920.	25529.1	{7855., 68134.}	67.8535	0.583341	newly 62 weeks cumul 85971. 26092.6 {8448., 68142.} 73.3213 0.719195
38	newly 3 weeks spec	14671.	987.86	{250., 1797.}	93.2666	1.36376	newly 3 weeks cumul	23153.	20806.8	{5529., 58365.}	75.0611	0.376317	newly 5 weeks spec	16782.	178.27	{4., 280.}	98.9377	2.66858	newly 5 weeks cumul	59731.	21333.	{6912., 58403.}	71.594	0.63296	newly 62 weeks cumul 80717. 22021.8 {8689., 58403.} 75.6174 0.727995
39	newly 3 weeks spec	8107.	1566.77	{58., 1639.}	90.5088	1.36475	newly 3 weeks cumul	18981.	10843.6	{708., 10479.}	94.1581	0.7558	newly 5 weeks spec	8590.	525.03	{2., 1041.}	93.8879	2.28407	newly 5 weeks cumul	36334.	12240.5	{749., 11095.}	90.9026	0.961426	newly 56 weeks cumul 64192. 13743.1 {749., 11384.} 88.6328 1.14605
40	newly 3 weeks spec	9375.	1306.45	{27., 1146.}	90.8455	1.35907	newly 3 weeks cumul	23924.	6132.44	{115., 4464.}	94.5652	1.10717	newly 5 weeks spec	8935.	610.45	{9., 777.}	93.1679	1.67153	newly 5 weeks cumul	42535.	7591.44	{147., 5645.}	91.7396	1.22856	newly 57 weeks cumul 74746. 10549. {169., 8798.} 89.1225 1.33574
41	newly 3 weeks spec	9002.	755.06	{17., 1429.}	91.6123	1.7121	newly 3 weeks cumul	21615.	5086.05	{169., 13573.}	87.8378	1.1875	newly 5 weeks spec	8585.	293.01	{0., 481.}	96.587	2.39349	newly 5 weeks cumul	39635.	5838.05	{236., 16097.}	86.7487	1.34875	newly 58 weeks cumul 64657. 6617.26 {394., 17610.} 90.3102 1.49646
42	newly 3 weeks spec	13145.	1274.05	{246., 2905.}	90.3077	1.24746	newly 3 weeks cumul	25907.	25684.7	{8488., 67727.}	81.7222	0.397754	newly 5 weeks spec	11817.	257.66	{6., 252.}	97.8196	2.45913	newly 5 weeks cumul	52038.	26446.	{8871., 68004.}	68.8996	0.551707	newly 62 weeks cumul 70332. 26864.3 {8878., 68011.} 70.0127 0.64203
43	newly 3 weeks spec	10255.	731.05	{0., 1063.}	95.8973	3.03755	newly 3 weeks cumul	22579.	10384.1	{611., 12506.}	80.7201	0.754439	newly 5 weeks spec	11209.	175.02	{0., 504.}	98.4386	3.38778	newly 5 weeks cumul	45260.	10885.2	{645., 12512.}	84.0467	0.97528	newly 55 weeks cumul 87509. 11501.1 {650., 12679.} 87.3429 1.1848
44	newly 3 weeks spec	15502.	233.6	{0., 550.}	98.4931	2.45868	newly 3 weeks cumul	33206.	7364.64	{1209., 17957.}	81.0781	0.868053	newly 5 weeks spec	12451.	44.84	{0., 91.}	99.6399	3.4373	newly 5 weeks cumul	61309.	7506.38	{1251., 17963.}	88.2021	1.11043	newly 61 weeks cumul 87960. 7569.2 {1260., 17963.} 91.3947 1.26026
45	newly 3 weeks spec	16177.	2744.37	{0., 5405.}	94.3647	2.37227	newly 3 weeks cumul	31598.	8609.59	{933., 45846.}	85.6458	1.07952	newly 5 weeks spec	13010.	1073.4	{0., 8917.}	91.7494	2.98709	newly 5 weeks cumul	61499.	11723.4	{933., 51538.}	85.7083	1.29342	newly 60 weeks cumul 80329. 12927.4 {939., 63079.} 84.2199 1.37226
46	newly 3 weeks spec	10769.	3304.59	{0., 18773.}	100.772	2.20589	newly 3 weeks cumul	20202.	9898.59	{921., 45213.}	93.8156	0.9389	newly 5 weeks spec	14108.	1266.	{0., 9146.}	91.0264	2.95203	newly 5 weeks cumul	48516.	13615.9	{926., 75022.}	86.8494	1.20269	newly 60 weeks cumul 82843. 14726.5 {932., 81016.} 82.7091 1.3626
47	newly 3 weeks spec	12425.	1373.83	{229., 3472.}	88.943	1.21309	newly 3 weeks cumul	24055.	24800.1	{6993., 67553.}	80.5566	0.376277	newly 5 weeks spec	13477.	303.09	{6., 305.}	97.7511	2.4716	newly 5 weeks cumul	52391.	25665.3	{8491., 67979.}	67.1099	0.545166	newly 62 weeks cumul 79732. 26193.1 {8863., 67985.} 71.6109 0.683257
48	newly 3 weeks spec	12536.	1242.06	{255., 2500.}	90.0921	1.20322	newly 3 weeks cumul	21158.	26063.3	{8756., 68190.}	93.6749	0.349282	newly 5 weeks spec	16548.	154.73	{4., 268.}	99.065	2.60824	newly 5 weeks cumul	54873.	26599.7	{8994., 68520.}	70.6452	0.563252	newly 62 weeks cumul 90499. 26861.4 {9006., 68527.} 74.6535 0.731072
49	newly 3 weeks spec	21183.	6673.82	{941., 20546.}	69.1357	0.75046	newly 3 weeks cumul	34718.	44954.9	{12432., 96229.}	82.106	0.320577	newly 5 weeks spec	14957.	1653.29	{74., 3660.}	88.9464	1.34292	newly 5 weeks cumul	71735.	49839.	{13880., 107104.}	58.9013	0.400192	newly 64 weeks cumul 87984. 52420.3 {14131., 107104.} 54.4119 0.407833
50	newly 3 weeks spec	10269.	974.43	{0., 986.}	96.6568	2.81948	newly 3 weeks cumul	26490.	8535.58	{980., 11569.}	85.906	0.863467	newly 5 weeks spec	9271.	265.69	{0., 716.}	97.1342	3.13267	newly 5 weeks cumul	46170.	9310.22	{980., 11569.}	87.0421	1.0338	newly 55 weeks cumul 81778. 10224.8 {980., 11690.} 88.2323 1.20357
